# Possible influences of vitamin D levels on sleep quality, depression, anxiety and physiological stress in patients with chronic obstructive pulmonary disease: a case control study

**DOI:** 10.5935/1984-0063.20210019

**Published:** 2022

**Authors:** Carlos José Dias, Rodrigo Barroso, Carlos Alberto Alves Dias-Filho, Andressa Coelho Ferreira, Christian Emmanuel Torres Cabido, Carlos C Crestani, Mayra Santos Silva, Alcimar Nunes Pinheiro, Bruno Rodrigues, Cristiano Teixeira Mostarda

**Affiliations:** 1 Universidade Federal do Maranhão, Departamento de Educação Física - São Luís - MA - Brazil.; 2 Universidade Estadual de São Paulo, Departamento de Farmácia - Araraquara - São Paulo - Brazil.; 3 Universidade Federal de São Paulo, Departamento de Psicobiologia - São Paulo - São Paulo - Brazil.; 4 Universidade Federal do Maranhão, Departamento de Medicina - São Luís -MA - Brazil.; 5 Universidade Estadual de Campinas, Departamento de Educação física -Campinas - São Paulo - Brazil.

**Keywords:** Anxiety, Depression, Sleep Quality, COPD, Vitamin D, Stress

## Abstract

This study aimed to evaluate the effect of vitamin D deficiency on sleep quality, depression, anxiety, and physiological stress in patients with chronic obstructive pulmonary disease (COPD). We screened for COPD patients with normal (NorVD) (n=24) and insufficient (InsVD) (n=7) vitamin D levels. The Pittsburgh sleep quality index (PSQI), the Beck anxiety inventory (BAI), the Beck depression inventory (BDI) and the Baevsky’s stress index were used for the sleep and psychometric evaluation. The evaluation of sleep quality by PSQI showed that NorVD individuals had higher duration and quality of sleep when compared with the InsVD group. Additionally, the group InsVD presented higher risk of developing sleep quality (OR=6.20; 95% CI=1.334, 29.013; p=0.009). BDI was higher in the InsVD, and this group had a higher risk of developing moderate and severe depression (OR=3.37; 95% CI=0.895, 12.722; p=0.03). The stress index indicated higher values in the InsVD in relation to the NorVD group (InsVD=24±0.8 vs. NorVD=16±0.9), and the group InsVD showed higher risk of developing high and very high physiological stress (OR=7.70; 95% CI=1.351, 43.878; p=0.01). The stress and sleep quality effects were negatively correlated with vitamin D levels. Insufficient levels of vitamin D negatively affect sleep quality and psychometric variables.

## INTRODUCTION

Chronic obstructive pulmonary disease (COPD) is a chronic disease strongly associated with severe morbidity and is currently the third leading cause of death globally^1^. Furthermore, poor indexes of quality of life are complications commonly observed in these patients due to conditions as depression, anxiety, stress, and worse sleep quality^2^.

Another relevant factor in COPD patients is vitamin D deficiency. Vitamin D deficiency is highly prevalent in a general population and has been associated with aggravating autoimmune diseases, allergies, endocrine and metabolic disorders, cancer, infections, cardiovascular diseases, low sleep quality and psychological disorders^3^. In COPD, vitamin D deficiency often occurs due to smoking-induced skin aging, reduced outdoor activity, and poor-quality food intake^4^.

The increased susceptibility to psychological stress and poor sleep quality related to vitamin D deficiency might play a vital role in the pathophysiology of depression and anxiety^5^. Accordingly, vitamin D receptors were identified in brain regions related to emotional-affective disorders such as the prefrontal cortex, hypothalamus and substantia nigra^6^.

The role of vitamin D in neuropsychiatric disorders seem to be mediated by its action as a neuroactive hormone involved in crucial functions, such as neuroprotection, neuroimmunomodulation, brain development, and regular brain function^7^. In this sense, vitamin D was demonstrated to increase the gene expression of factors regulating the production of serotonin, dopamine, and a nerve growth factor^6,8^. Therefore, it is suggested that the maintenance of normal vitamin D levels plays a fundamental role in mental health^8^.

The maintenance of mental health has received more and more attention and has been an important focus of study by health professionals as it affects the general quality of life and chronic distress^1^. Mental disorders compromise not only the quality of life of COPD patients but impose a critical economic burden, as they are important risk factors for several other diseases^9^.

Although patients with COPD are more likely to develop depression, anxiety, stress, and poor sleep quality, it is not completely clear whether vitamin D deficiency in these patients may interfere with these variables. Therefore, since vitamin D deficiency appears to be common in COPD patients and its levels seems to be related to mental health and quality of life in the general population, this study investigated the effect of serum vitamin D levels on sleep quality, stress, depression, and anxiety in COPD patients.

## MATERIAL AND METHODs

### Participants

This was a cross-sectional study of COPD patients from the Outpatient Clinic of the Asthmatic Patient Assistance Program of the Maranhão Federal University Hospital. The sample consisted of 39 COPD patients. A sample error of 5% and a confidence interval of 95% were adopted. The COPD patients were divided into two groups: insufficient vitamin D (InsVD) and normal vitamin D (NorVD).

Patients were excluded if they had any of the following conditions: having other nutritional problems besides vitamin D deficit, a history of hypercalcemia, cancer, presence of pulmonary infection, tuberculosis, pleural effusion, congestive heart failure, primary pulmonary hypertension, pulmonary embolism, restrictive airway disease, vitamin D supplementation, orthopedic disorders affecting physical performance, and inability to walk.

## EXPERIMENTAL PROCEDURES

### Anthropometric evaluation

Weight measurement was performed using a digital scale on the kilogram scale (Balmak, BK - 50FAN, São Paulo, SP, Brazil). For stature, the EST 23 Trena Compact Stadiometer was used in the millimeter scale. Also, the body mass index (BMI) was calculated using the formula weight (kg)/height (m^2^).

### Blood pressure measurements

The procedures for the measurement of blood pressure (BP) were made according to the guidelines of the seventh report of the Joint National Committee on Prevention, Detection, Evaluation, and Treatment of High Blood Pressure (JNC7)^9^. In summary, patients remained in the sitting position in a comfortable chair for 20 min. With an automatic and non-invasive BP monitor (BP710, Omron, Tokyo, Japan), three measurements of BP were performed on the right arm, with at least a 2-min interval between each one.

### Respiratory function

One morning was reserved for data collection of spirometry tests (MicroLoop Spirometer, CareFusion, Yorba Linda, CA, U.S.) by a qualified technician. Subjects underwent the spirometry test in the sitting position, wearing a nose clip to obtain the following parameters: forced vital capacity (FVC); forced expiratory volume in 1sec (FEV1); as well as the ratio of FEV1 to FVC (FEV1/FVC, expressed as a percentage), and all his predicted values according to the American Thoracic Society (ATS)^7^.

In addition to the automatic evaluation, the quality of spirometric tests was assessed according to some criteria, including the number of acceptable maneuvers according to American Thoracic Society, ranging from 0 to 3, the highest kept by the spirometry software; the reproducibility (FEV1 and FVC were considered reproducible according to ATS criteria when the best two trials differed by not more than 200mL).

### Measurement of physiological stress

Physiological stress was evaluated by heart rate variability (R-R intervals), using a 12-lead electrocardiogram, with a sampling frequency of the electrocardiogram signal of 600Hz (Micromed Biotecnologia® Ltda., Brasília, DF, Brazil), and obtaining moment by moment R-R intervals. The subjects remained lied down for at least 20 minutes before the recording onset, and the electrocardiogram was monitored for 10 minutes.

Then, the series of R-R intervals were extracted in *.txt* through the same analysis software (Wincardio® 6.1.1 Ltda., Brasília, DF, Brazil) for analysis of the heart rate (HR) variability of the R-R interval in the time domain. The index used was mean R-R, mean HR, RMSSD, SD1 (%), and SD2 (%). The stress index was the square root (to make the index normally distributed) of the Baevsky’s stress index^17^. The Baevsky’s stress index values between 50-150 are considered normal, 150-500 elevated, 500-900 high, and >900 very high^10^.

### Serum vitamin D level

Serum vitamin D level was extracted from a small blood sample and evaluated by measuring 25-hydroxyvitamin D (25-OH-D) using the chemiluminescence method according to the manufacturer’s instructions. Serum 25-OH-D levels <20ng/mL was considered deficient, from 20 to 30ng/mL was considered insufficient levels (InsVD) and ≥30ng/mL was appropriate values (NorVD). The analysis was performed in the laboratory of the Maranhão Federal University Hospital.

### Sleep quality, anxiety, and depression assessment

The quality of sleep and the presence of sleep disorders were evaluated using the Pittsburgh sleep quality index (PSQI), as initially described by Buysse^14^. The PSQI used seven components: i) subjective sleep quality, ii) sleep latency, iii) duration of sleep, iv) habitual sleep efficiency; v) sleep disorders; vi) use of medication for sleep; and vii) daytime sleepiness and disorders during the day. Each element was individually determined and the score of each component was added to give an overall score ranging from 0 to 21 points. The higher the value obtained, the worse the quality of sleep (global score is between 6 and 21). For good sleep quality, the sum of the scores is only 5.

The Beck anxiety inventory (BAI) and the Beck depression inventory (BDI) were used to evaluate anxiety and depression levels, respectively, as initially described by Beck^11^. BAI is a 21-item checklist developed with large clinical samples to measure anxiety symptoms according to the fourth edition of the *Diagnostic and Statistical Manual of Mental Disorders* of the American Psychiatric Association (APA)^12^. Application and scoring are rapid and the patient rates symptom intensity during the last seven days on a scale from 0 (not at all) to 3 (severely). The BAI adequately covers the significant cognitive, affective, and physiological symptoms of anxiety^13^.

To verify the presence of self-reported depression, patients answered the BDI and minimal, mild, moderate, and severe scores for major depressive episodes were obtained, according to the APA. The BDI is scored by summing the highest ratings for each of the 21 items. Each item is rated on a 4-point scale ranging from 0 to 3, and the total scores can range from 0 to 63. BDI total scores ranging from 0 to 13 represent “minimal” depression; total scores from 14 to 19 are “mild;” total scores from 20 to 28 are “moderate;” and total scores from 29 to 63 are “severe”^11,12^.

### Statistical analysis

Data were reported as mean±standard deviation. The normality of data was tested using the Kolmogorov-Smirnov test. An unpaired t-test was used to compare parametric data. The chi-square was used to compare the groups when data were distributed in frequency. Pearson correlation was used in parametric data. Differences were considered significant when *p*<0.05. We used GraphPad Prism 5 for statistical analysis. Effect size higher than 0.8 were considered very strong; values ranging from 0.6-0.8 were considered strong; values ranging from 0.4-0.6 were considered moderate; and lower than 0.4 were considered negligible^11^.

## RESULTS

Blood pressure, spirometry test and body composition variables in COPD individuals with normal and insufficient vitamin D.

As presented in the Table 1, no differences were observed between the groups in the height, weight, and BMI. Additionally, we did not find statistical difference in the hemodynamic variables systolic blood pressure (SBP) and diastolic blood pressure (DBP).

**Table 1:** Vitamin D, blood pressure, spirometry test, and body composition variables in COPD individuals with normal (NorVD) and insufficient (InsVD) vitamin D.

	NorVD (n=24)	InsVD (n=17)	p	ES
Vitamin D (ng/ml)	41±7.9	26±2.4	0.0001	2.56
**Spirometry test**				
FVC (L)	2.12 ± 0.69	1.65±0.62	0.10	0.71
FEV1 (L)	1.26±0.53	1.13±0.45	0.55	0.26
FEV1/FVC (%)	61±20	66±24	0.77	0.22
FEF 25-75%	0.73±0.47	0.58±0.35	0.40	0.36
**Body composition**				
Age (years)	72.43±11.05	72.73±10.43	0.94	-0.09
Weight (kg)	64.25±16.20	56.33±9.72	0.11	0.59
Height (m)	158.35±7.58	156.22±6.83	0.46	0.28
BMI(Kg/m^2^)	25.44±5.17	23.05±3.41	0.15	0.46
Systolic blood pressure (mmHg)	146±23	148±16	0.60	0.10
Diastolic blood pressure (mmHg)	80±12	79±10	0.59	0.09

Notes: Values are presented as mean±standard deviation; unpaired t-test; effect size values higher than 0.8 were considered very strong; values ranging from 0.6-0.8 were considered strong; values ranging from 0.4-0.6 were considered moderate; and lower than 0.4 were considered negligible.

The assessment of pulmonary function indicated that the NorVD group showed similar values of FVC (forced vital capacity, L) and FEV (forced expiratory volume, L) when compared to the InsVD group. Additionally, there was no significant difference in FEV1/FVC and FEF 25-75 % (Table 1).

### Sleep quality in COPD individuals with normal and insufficient vitamin D

The evaluation of sleep quality by PSQI showed that NorVD individuals had a higher duration of sleep and quality of sleep when compared to the InsVD group (Table 2). Additionally, the InsVD group showed a higher risk of developing sleep disorders (OR=6.20; 95% CI=1.334, 29.013; *p*=0.009). Subjective sleep quality, sleep latency, sleep efficiency, sleep disorders, use of sleeping medication, and daytime sleepiness were similar (Table 2).

**Table 2:** Sleep quality in COPD individuals with normal (NorVD) and insufficient (InsVD) vitamin D.

		NorVD (n=24)		InsVD (n=17)		p
	Category	N	%	N	%	
**Subjective sleep quality**	Very good	1	4%	1	6%	0.56
	Good	17	71%	9	53%	
	Bad	4	17%	6	35%	
	Very bad	2	8%	1	6%	
**Sleep latency (min)**	≤15	13	54%	5	29%	0.29
	16 a 30	1	4%	1	6%	
	31 a 60	6	25%	4	24%	
	> 60	4	17%	7	41%	
**Duration of sleep (hours)**	> 7	12	50%	1	6%	0.02
	6-7	6	25%	6	35%	
	5-6	4	17%	6	35%	
	< 5	2	8%	4	24%	
**Sleep efficiency (%)**	> 85	11	46%	5	29%	0.66
	75-84	8	33%	6	35%	
	65-74	2	8%	3	18%	
	<65	3	13%	3	18%	
**Sleep disorders (events/weeks)**	None	1	4%	0	0%	0.56
	< 1	8	33%	4	24%	
	1-2	13	54%	8	47%	
	3	2	8%	4	24%	
**Use of sleeping medication (events/weeks)**	None	20	83%	14	82%	0.85
	<1	2	8%	0	0%	
	1-2	0	0%	0	0%	
	3	2	8%	3	18%	
**Daytime sleepiness**	None	8	33%	3	18%	0.31
	Small	8	33%	4	24%	
	Moderate	7	29%	7	41%	
	High	1	4%	3	18%	
**Sleep quality**	Good (0-4)	5	21%	0	0%	0.04
	Bad (5-10)	16	67%	9	53%	
	Disturb (>10)	3	13%	8	47%	

**Notes:** Results are presented by distribution frequency and relative percentage; chi-square test.

### Anxiety, depression, and stress index in COPD individuals with normal and insufficient vitamin D

We present in the Table 3 the anxiety and depression scores assessed by the Beck inventory. The anxiety index (BAI) did not indicate statistical difference between NorVD and InsVD groups, and both groups presented minimum levels of anxiety. However, the depression index (BDI) was higher in the InsVD group in relation to the NorVD group. Additionally, the group InsVD showed a higher risk of developing moderate and severe depression (OR=3.37; 95% CI=0.895, 12.722; *p*=0.03).

**Table 3:** Anxiety (BAI) and depression (BDI) results in COPD individuals with normal (NorVD) and insufficient (InsVD) vitamin D.

		NorVD (n= 24)		InsVD (n=17)		p
	Category	N	%	N	%	
**BAI**	Minimum (0-10)	16	66.67	9	52.94	0.53
	Mild (11-19)	4	16.67	3	17.65	
	Moderate (20-30)	2	8.33	1	5.88	
	Severe (31-63)	2	8.33	4	23.53	
**BDI**	Minimal (0-9)	10	41.67	3	17.65	0.03
	Mild (10-18)	8	33.33	5	29.41	
	Moderate (19-29)	4	16.67	1	5.88	
	Severe (30-63)	2	8.33	8	47.06	

**Notes:** Results are presented by distribution frequency and relative percentage; chi-square test.

The stress index was higher in the InsVD group in relation to the NorVD group (Table 4). The data showed a modification in levels of physiological stress categories normal (25% vs. 6%), high (8% vs. 35%), and very high (0% vs. 6%) (*p*=0.0007) (Table 4). Additionally, the group InsVD showed higher risk of developing high and very high physiological stress (OR=7.70; 95% CI=1.351, 43.878; *p*=0.01).

**Table 4:** Stress index in COPD individual with normal (NorVD) and insufficient (InsVD) vitamin D.

		NorVD (n= 24)		InsVD (n=17)		p
	Category	N	%	N	%	
**STRESS INDEX**	Low (<7)	0	0	0	0	0.007
	Normal (7-12)	6	25	1	6	
	Elevated (13-22)	16	67	9	53	
	High (23-30)	2	8	6	35	
	Very high (>30)	0	0	1	6	

**Notes:** Results are presented by distribution frequency and relative percentage; chi-square test.

**Figure 1:**
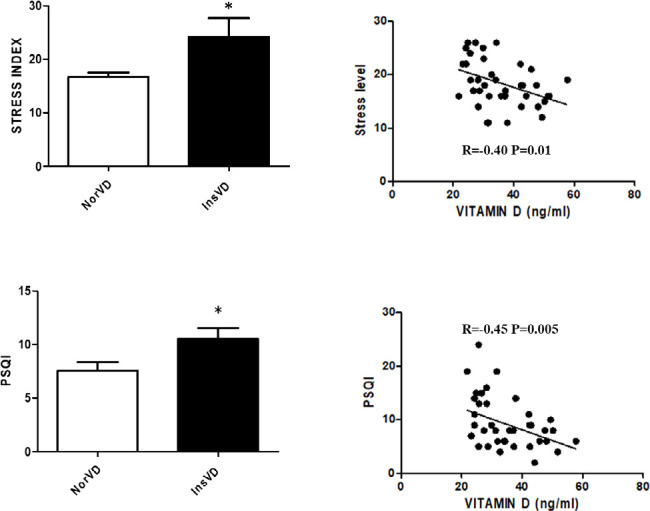
Correlation of stress (top graphs) and sleep quality (PSQI, bottom graphs) indexes and levels of vitamin D in COPD individuals.

Correlation analysis indicated the stress and sleep quality indexes negatively corrected with vitamin D levels, suggesting that individuals with lower levels of vitamin D present higher levels of stress and impaired sleep quality (Figure 1).

## DISCUSSION

This study aimed to investigate the effect of serum vitamin D levels on sleep quality, depression, anxiety, and physiological stress in COPD patients. The main findings of the present study are that insufficient vitamin D levels in patients with COPF evokes impaired sleep quality, depression, and higher levels of physiological stress. Additionally, stress index and sleep quality negatively correlated with vitamin D levels.

These data corroborate with other studies that have shown a relationship between sleep quality and vitamin D levels. For instance, a meta-analysis discussed the relationship between vitamin D deficiency and sleep disorders, including poor sleep quality, short sleep duration, and drowsiness. Accordingly, low serum 25-(OH)-D levels has been reported to be associated with an increased risk of sleep disorders^14^. Besides, vitamin D supplementation improved sleep latency and sleep duration in subjects with vitamin D deficiency^15^. However, these studies did not investigate patients with COPD, so that present study provide the first evidence that this relationship is also observed in patients susceptible to sleep disorders.

In addition to changes in sleep quality, we observed a higher score in depression questionaries’ (BDI) in COPD patients with insufficient vitamin D levels. Interest in role of vitamin D in depression has increased. This is because of evidence of increased expression of vitamin D receptors (VDRs) in brain areas that play a crucial role in regulating mood^16^. Besides, adequate levels of vitamin D can act as a modulator of inflammatory response, thus presenting neuroprotective actions^17,18^. Additionally, vitamin D regulates the production of serotonin, dopamine, and a nerve growth factor in the brain. These substances have been prominent in the prophylaxis and therapy of psychiatric disorders^19^. Supporting these findings, several studies have identified vitamin D deficiency as an essential factor for depression^20-22^. However, these previous studies have not explored the interactions between stress, depression, anxiety, and sleep quality in patients with COPD.

Another variable evaluated in this study was physiological measures of stress by the Baevsky stress index. The InsVD group showed higher stress levels than NorVD groups. This finding is possibly related to the impaired sleep quality and increased levels of depression. In fact, the stress has been documented as a prominent environmental factor involved in etiology and development of mood disorders^23,24^. Furthermore, although we did not find changes in blood pressure, elevated psychosocial stress can contribute to cardiovascular disease as endothelial dysfunction, atherosclerosis, and impaired autonomic control^25,26^.

This idea is supported by the fact that the Baevsky stress index is strongly linked to increased cardiac sympathetic response during stressful situations. Also, some authors used this method to assess the level of stress along with psychometric variables^27^ and autonomic dysfunction has been recognized as a significant predictor of mortality. Additionally, vitamin D supplementation is associated with improved autonomic modulation in healthy humans^28^. Therefore, adequate levels of vitamin D can be crucial to cardiovascular health, preventing endothelial cell death in human cells, inhibiting the generation of superoxide anion, and maintaining mitochondrial function^21^.

Although studies have suggested that psychological variables are associated with sleep disorders and low levels of vitamin D can lead to chronic physiological stress, few reports investigated this relationship in patients with COPD. In this sense, the identification of population subgroups that have increased level of depression and, perhaps, have a more significant benefit in vitamin D supplementation is a relevant aspect.

We believe that this work will encourage professionals to consider the relationship between low levels of vitamin D and depression, sleep quality, and stress when treating patients with COPD. However, further studies are necessary to standardize dose, frequency, and duration of supplementation or administration of vitamin D in these patients. Besides, the association of changes in vitamin D levels with inflammatory markers over time as well as adjust confounding factors like lifestyle and demographic factors are issues to be addressed in further studies.

## CONCLUSION

We conclude that levels of vitamin D are negatively correlated with sleep quality, depression, and stress in patients with COPD.
